# Editorial: Plant genetic and genomic resources for sustained crop improvement

**DOI:** 10.3389/fpls.2023.1266698

**Published:** 2023-08-10

**Authors:** Parimalan Rangan, Robert Henry, Peterson Wambugu, Sambasivam Periyannan

**Affiliations:** ^1^ Division of Genomic Resources, Indian Council of Agricultural Research (ICAR)-National Bureau of Plant Genetic Resources, New Delhi, India; ^2^ Queensland Alliance for Agriculture and Food Innovation, The University of Queensland, St Lucia, QLD, Australia; ^3^ Kenya Agricultural and Livestock Research Organization, Genetic Resources Research Institute, Nairobi, Kenya; ^4^ School of Agriculture and Environmental Science & Centre for Crop Health, University of Southern Queensland, Toowoomba, QLD, Australia

**Keywords:** plant genetic resources (PGR), domestication, genomics, next generation (deep) sequencing (NGS), crop improvement

The center of origin for a crop has, in general, been associated with the representation of the maximal extent of its diversity. However, one should also be cautious that a crop can develop multiple centers of diversity during the path of domestication and cultivation (Harlan, 1971; Harlan, 1975). The proposed protracted multiple-centric model for the domestication process holds good with special reference to cultivated crops and to lesser extent for its wild relatives due to the higher artificial selection pressure in cultivated ones while only natural selection pressure has been applied to wild relatives (Allaby et al., 2008). This is reflected in the co-existence of diverse traits in different germplasm accessions of a crop, in an array pattern, at multiple locations with each accession possessing different combinations of traits of interest (Esquinas-Alcázar, 2005). For example, to represent complete variability for the traits like grain size and color, plant architecture, seed shattering (but amenable to threshing), various abiotic and biotic stress tolerance, glutinous grain, flowering time, and life cycle (short-, medium-, and long-duration) in rice, we require many numbers of genotypes (Izawa, 2022; Shang et al., 2022). If we tabulate the outcome of selection pressure during domestication process in the form of trait versus variability with each cell containing appropriate genotype(s), what we obtain is an array pattern of germplasm representing diverse phenotypic traits and the variability within. This will reveal, crop plants are more prone to genetic erosion (compared to crop wild relatives, CWRs) if a particular genotype is lost. This is due to the fact that CWRs weren’t diversified (in array pattern) due to lack of artificial selection pressure although natural selection pressure did exist. Conserving such precious crop genetic resources and CWRs are very crucial for food security through sustained crop improvement.

Next-generation sequencing (NGS) technologies have transformed genome sequencing – from an effort involving multi-national and multi-institutional consortia working over decades to something that can be accomplished in a few months in a single lab or a team within an institute. Genomic resources and sequence information have led to a better understanding on the genetic architecture of crop plants with special reference to the steps of domestication involving transcription factor loci at first and followed by targeted selection of enzyme-coding genes (Purugganan and Fuller, 2009; Meyer and Purugganan, 2013). Comparative genomics have also helped identify the existence of parallel evolution for key loci involved in domestication events. For example, mutation in the *waxy* gene coding for granule-bound starch synthase (GBSS) enzyme underwent targeted selection during domestication in rice, sorghum, broomcorn millet, foxtail millet, barley, and amaranths, producing sticky glutinous grain, when boiled, due to reduced amylose levels. Likewise, other domestication loci such as the *Fw2.2* gene for fruit weight, *Prog1* gene for erect-plant phenotype, *Rc* gene for anthocyanin content, and *Sh* gene for shattering are also known to have been involved in parallel domestication events in multiple crops (Meyer and Purugganan, 2013).

Lee et al. utilized rice genetic and genomic resources to map two quantitative trait loci (QTLs), *qPH2* and *qPH7*, associated with the pre-harvest sprouting (PHS) trait and these two QTLs explain 38% of the phenotypic variation. These were identified through the development of a recombinant-inbred mapping population (F_8_) of 186 lines derived using Hwayeong, a *japonica* rice cultivar (PHS sensitive) and a japonica-type weedy rice tolerant to PHS and use of a 7K Infinium SNP genotyping platform and construction of a linkage map. In a similar approach using a mapping population in spinach (*Spinacia oleracea*), Bhattarai et al. developed near-isogenic lines by crossing the downy mildew resistant cultivar Whale with the susceptible cultivar Viroflay and identified the NBS-LRR gene, *SOV3g001250*, associated with the resistance. Whole genome resequencing approaches were used at low coverage to identify the SNP markers associated with the resistance using genome-wide association studies. Genomic selection models were used to validate the SNPs identified. Three SNP markers were identified for future use in molecular breeding to incorporate downy mildew resistance in spinach.

Ibrahim et al. studied 327 rapeseed (*Brassica napus* L.) accessions variable for shoot fresh weight and potassium utilization index traits and identified key regulatory genes associated with potassium utilization efficiency (KUE) using RNA-seq of 20 accessions contrasting for KUE. They identified three candidates, *BnaC04G0560400ZS*, *BnaC04G0560400ZS*, and *BnaA03G0073500ZS* that were differentially regulated between the high and low KUE accessions.

Genomic resources generated in various species have been applied to identifying the genes underlying the traits of interest for use in trait-based-genetics for molecular breeding purposes. Shah et al. generated whole genome sequence (WGS) from the leaves of seven weed species (*Alternanthera philoxeroides*, *Lycium ferocissimum*, *Senecio madagascariensis*, *Lantana camara*, *Parthenium hysterophorus*, *Cryptostegia grandiflora*, and *Eichhornia crassipes*) and studied key herbicide-target genes. Although the authors noted the acetolactate synthase (*als*) and 5-enolpyruvylshikimate-3-phosphate synthase (*epsp*) genes were conserved, specific mutations in the *als* gene of *S. madagascariensis* and *P. hysterophorus* were found to provide resistance to ALS-inhibition by herbicides. These are potential target sites for developing novel herbicides in the future and can act as a new source of genes for use to develop transgenic crop plants for herbicide tolerance. Similarly, Achakkagari et al. used a *de novo* WGS strategy and sequenced a panel of nine diploid potato (*Solanum tuberosum* L.) clones, with genome sizes ranging between 712 to 948 Mbp, and studied the genetics for earliness of the tuberization trait. The sequence information from these nine clones helped the authors identify novel variants for the *StCdf1* gene (associated with the earliness of the tuberization trait). In another WGS study, Gaikwad et al. generated a draft genome for small cardamom (*Elettaria cardamomum* Maton. cv. Njallani Green Gold) with a genome size of 1.06 Gbp and 68,055 genes predicted. A total of 250,571 simple sequence repeat (SSR) markers were identified.

In maize, the genetics of haploid induction and other agronomic traits were determined by Dermail et al. using eight maize genotypes with six inbreds of haploid inducers (KHI42, KHI47, KHI49, KHI54, and KHI59 tropical types) and BHI306 (a temperate type), and two haploid non-inducers (hybrid S7328 and an inbred Takfa1) in an 8x8 full diallel mating design. Anthocyanin pigmentation was used as a marker to distinguish the diploid and haploid seeds. Their study identified BHI306 genotype as the best combiner for *in vivo* haploid induction. Dossa et al. underscored the importance of genetic and genomic resources in crop improvement with special reference to maize for developing striga resistant cultivars, since maize is exceptionally susceptible. This review also highlighted the various strategies to utilize advanced genomics-assisted breeding tools like genomic selection, genome-wide association, marker-assisted breeding, and genome editing approaches to overcome striga problem.

Overall, this Research Topic demonstrates the synergy in addressing researchable issues that contribute towards enhanced crop productivity through a combination of genetic and genomic resources. Until the end of 20^th^ century, although diverse crop genetic resources were conserved in Genebanks, they couldn’t be used efficiently in crop improvement programs (Hoisington et al., 1999). Due to lack of genomic resources, there existed a gap in identifying the promising accessions that could be used for introgression of desired traits to develop high yielding cultivars with durable biotic and abiotic resistance without reduction in quality traits. Capacities to generate genomic resources at affordable cost have bridged this gap as evident from this set of articles representatively utilizing various genomic and genetic resources for crop improvement.

## Author contributions

PR: Conceptualization, Writing – original draft, Writing – review & editing. RH: Writing – review & editing. PW: Writing – review & editing. SP: Writing – review & editing.
